# Relationship between Precarious Employment and Unmet Dental Care Needs among Korean Workers: A Longitudinal Panel Study

**DOI:** 10.3390/medicina58111547

**Published:** 2022-10-28

**Authors:** Xianhua Che, Minsung Sohn, Sungje Moon, Hee-Jung Park

**Affiliations:** 1 Department of Health Policy Research, Daejeon Metropolitan City Public Health Policy Institute, Daejeon 35015, Korea; 2 Division of Health and Medical Sciences, The Cyber University of Korea, Seoul 03051, Korea; 3 Research Institute for Healthcare Policy, Korean Medical Association, Seoul 04373, Korea; 4 Department of Dental Hygiene, Kangwon National University, Samcheok 25945, Korea

**Keywords:** barriers to access, dental care, health services, precarious employment, workforce

## Abstract

*Background and Objectives*: Precarious workers experience certain conditions, such as low income, instability in employment, and lack of social security. Precarious employment has increased barriers to the use of dental care services, resulting in more unmet dental care needs. The aim of this study was to identify unmet dental care needs among precarious workers in Korea’s labor market, using data from the Korea Health Panel Survey (2011–2017). *Materials and Methods*: Based on job and income security criteria, four groups were formed: Group A (individuals with job and income security), Group B (individuals reporting job security with income insecurity), Group C (individuals reporting job insecurity with income security), and Group D (individuals with job and income insecurity). We measured self-reported unmet dental need or the inability to receive necessary dental care owing to the past economic burdens. Panel logistic regression analyses were performed to determine the effect of precarious employment on unmet dental care needs for all participants. *Results*: Approximately 16% of the respondents reported having unmet dental care needs. Unmet dental care needs owing to economic reasons were higher among male workers in groups C and D than among male workers in Group A. In particular, male workers aged 50 years and above in Group B were 3.36 times more likely to have unmet dental care needs than those in Group A. In Group D, female workers showed a high probability of having dental care needs owing to economic reasons. Moreover, female workers aged 18–49 years witnessed an increase in unmet dental care needs. *Conclusions*: Korean workers with unstable employment and/or income are at a higher risk of having unmet dental care needs owing to financial factors. The findings suggest an urgent need to implement robust national health insurance policies to improve efforts aimed at reducing unmet dental care needs that potentially decreases the disparity in oral health among precariously employed workers. Furthermore, it is necessary to implement comprehensive labor market policies such as sickness benefits for those in precarious employment.

## 1. Introduction

In recent decades, the flexibilization of the labor market has become a global trend driven by globalization and international technological competition [1]. This trend has led to rapid changes in the workplace environment and increased the precariousness of employment. Precarious work has been defined as the combination of job insecurity, loss of control over work, low levels of protection (prevention of unemployment or discrimination), and lack of opportunities for training and career advancement [2]. In the context of Korea, companies have been actively pursuing their flexibility through layoffs, outsourcing, and non-regular employment, mainly since the Asian Financial Crisis in 1997 [3]. These trends have placed South Korea at the topmost position among Organization for Economic Co-Operation and Development (OECD) countries with precarious workers [4]. As of 2020, more than 26.1% of the total economically active population (labor force) represents temporary workers, that is much higher than the OECD average of 11.4% [5]. Specifically, the proportion of self-employed workers in the Korean labor market is higher than that of the other OECD countries, and a sizeable number of self-employed workers have no or few employees (less than four).

Evidence indicates that precarious employment is characterized by insecurity, instability, and hazardous working conditions, among others, that adversely impact several precariously employed workers [6]. Precarious workers also suffer because of economic burden and lack of social benefits (i.e., low earnings, paid sick leave, and limited or no insurance and pensions) accompanying unstable employment [7,8]. Studies have indicated that precarious workers are at a greater risk of occupational diseases, injuries, and premature death than those holding full-time or standard jobs [9,10]. For this reason, the increase in precarious workers also exacerbates major health inequalities [10].

The poor health outcomes of precarious workers are associated with factors such as smoking, drinking, unhealthy eating habits, and worsening psychosocial and physiological conditions owing to occupational risks (primarily job insecurity and poor working conditions) [11,12]. These health and employment patterns might also reflect the disparities in access to and use of healthcare, resulting in unmet healthcare needs [4]. A growing body of literature has provided evidence that insecure employment and poorer working conditions are associated with oral illness and limited access to dental services [13]. A previous study indicated that workers with precarious employment have a high tendency to forgo dental care [14]. Notably, precarious workers with a relatively low socioeconomic status lack health insurance, that potentially increases the disparity in the use of dental services. Concerning the frequency of healthcare, a study found that the incidence of unmet dental care needs among irregular workers is 1.69 times more than that of regular workers [15]. Another study verified that precarious workers are more likely to have limited access to dental care and unmet dental care needs than their non-precarious counterparts [16].

Unlike other countries, South Korea achieved universal health insurance coverage after the implementation of the National Health Insurance (NHI) system in 1989. Therefore, Koreans can subscribe to NHI whether they work or not. Despite this, a relatively low health insurance coverage may result in unmet healthcare needs, that is more likely to occur in the case of dental care since it often requires higher out-of-pocket (OOP) payments than other regular healthcare services [15,16]. Particularly, dental care currently accounts for approximately 20% of dental service expenditures of public programs in South Korea. Such barriers to accessing dental services increase the rate of unmet dental care needs among low-income precarious workers [15].

The age group was classified based on the age of 50, considering that the average retirement age from the first major job of Korean workers suggested by the national statistical office was 49.3 years old, and the previous studies on the jobs and retirement of middle-aged and elderly individuals in Korea were conducted based on the age of 50 [17]. Previous studies have suggested that the unmet health care needs were different according to the difference in employment types between men and women in the labor market [17,18]. Based on the results of our previous study, to provide effective measures for improving dental care accessibility, it is necessary to examine whether precarious workers’ unmet dental care needs differ according to gender and age group [19]. Given the impact of age and gender on the labor market, it is also possible that improving precarious workers’ access to dental care services in consideration of type of work by gender and age. Therefore, we examine the level of access to necessary dental care services based on gender and age in precarious and non-precarious workers using representative sample data from South Korea.

## 2. Materials and Methods

### 2.1. Data Source and Study Population

In this study, we used data from the Korea Health Panel Survey (KHPS), that has been comprehensively monitoring patients’ medical usage, medical expenses, and associated socioeconomic factors since 2008. Our study can be seen as an extension of the study conducted in 2018 [19]. The study conducted in 2018 used one-year data from 2015. On the other hand, the current study performed a panel analysis using seven-year data from 2011 to 2017.

**Figure 1:**
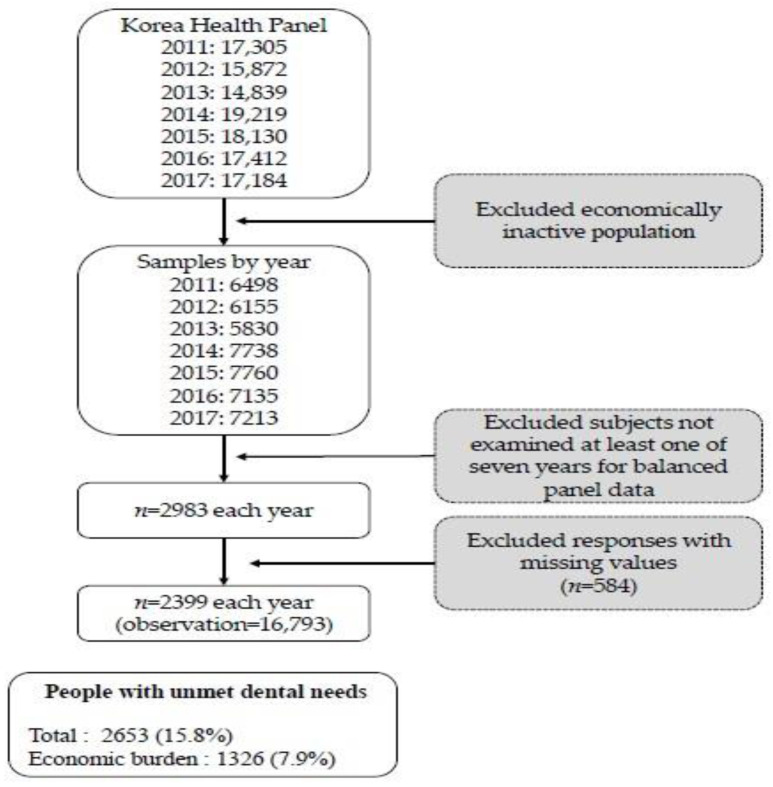
The flow of sample selection in this study.

Based on the longitudinal data, a survey was conducted among economically active individuals aged over 18 years to examine their experience of unmet dental care needs, employment, and income status. Given that the survey has been measuring unmet dental care needs since the 6th wave, a total of 2399 people who answered related questions every year between 2011 and 2017 were selected for the present study. Specifically, 2653 out of 16,793 people in total answered questions on unmet dental care needs (sum up to 7 years) [Figure 1].

### 2.2. Measurement

#### 2.2.1. Independent Variable

In this study, we considered precarious employment as the independent variable, measured by employment relationship and income insecurity. First, employment status was classified as precarious if any of the following criteria was unstable: type of employment contract (temporary, daily, or self-supporting position/periodic contract); working hours (part-time workers); and working relationship (indirect or special employment). Non-wage workers, self-employed workers with less than four employees, and unpaid family workers were also included under precarious employment. Second, income insecurity was defined as per the International Labor Office’s low-wage standards, which define low-wage workers as those earning less than two-thirds of the median income of all workers. A similar standard was used for non-wage workers [19].

Based on the aforementioned employment and income insecurity criteria, the following groups were formed: (1) Group A, comprising individuals with both employment and income security (e.g., permanent workers); (2) Group B, comprising individuals reporting job security with income insecurity (e.g., workers involved in cleaning, facility management, security, parking management, call centers, and public sectors); (3) Group C, comprising individuals experiencing job insecurity and income security (e.g., temporary or daily wage workers in construction sites, freelance professionals, and courier workers); and (4) Group D, comprising individuals with job and income insecurity (e.g., housekeepers, learning tutors, small-scale self-employed individuals with no or less than four employees) (Table 1).

#### 2.2.2. Dependent Variable

For this study, we used the occurrence of unmet dental care needs as the dependent variable. Respondents stating, “I have not received necessary dental treatment at least once in the past year” were considered to have experienced unmet dental care needs and coded as “1.” We also hypothesized economic factor to be the most important reason for not receiving the necessary dental treatment.

#### 2.2.3. Covariates

The covariates comprised the demographic characteristics and health status of the study population. The demographic characteristics included marital status (1: Married, 2: Divorced/Widowed/Separated, and 3: Single); education level (1: More than 12 years and 2: Less than 12 years); and type of health insurance (1: Health insurance employer, 2: Health insurance local subscriber, and 3: Medical aid). We also measured the national insurance subscription (1: Yes and 2: No), that included subscribers enrolled in any of the four insurances (national pension, NHI, employment insurance, and industrial accident insurance), and private health insurance (1: Yes and 2: No). Self-rated health status (1: Good, 2: Moderate, and 3: Poor) and disability (1: None and 2: Present) were used as health status variables.

#### 2.2.4. Statistical Analyses

The statistical analyses were conducted using STATA/BE 17.0 (TX: StataCorp LLC). All data were stratified by gender (1: Male and 2: Female) and age (1: Below 50 years [18–49 years] and 2: Over 50 years). First, we described respondents’ general characteristics and determined the prevalence of unmet dental care needs. Second, we used a chi-square test to assess the differences in the total unmet dental care needs based on demographic characteristics. We conducted a time-series analysis to present the frequency of unmet dental care needs by gender and age group every year. Third, a panel logistic regression analysis was performed to determine unmet dental care needs and unmet dental care needs due to economic burden by precarious employment.

Ethical review and approval were waived for our study, due to that we used publicly open data without personal identification.

## 3. Results

### 3.1. Participant Characteristics

Table 2 presents the general characteristics of the study population. First, concerning the four groups, Group A accounted for the highest number of participants at 35.2%, followed by groups C, D, and B at 35.0%, 26.2%, and 3.6%, respectively. Married persons and workers with less than 12 years of education accounted for 83.2% and 63.0% of the total study population, respectively. The economically active participants who did not subscribe to any of the four major insurances accounted for 57.4% of the population. Concerning the type of health insurance, NHI beneficiaries among employees and self-employed of the total sample accounted for 70.2% and 29.0%, respectively. Private insurance subscribers represented the majority of the study population at 85.7%. Self-rated health status was found to be good, normal, and poor for 46.6%, 45.3%, and 8.1% of the population, respectively. Moreover, 96.1% of the population had no disabilities.

The economically active population experiencing unmet dental care needs represented 15.8% of the total sample. Of this percentage, the unmet dental care needs of 7.9% of the population were attributed to economic factors.

**Figure 2:**
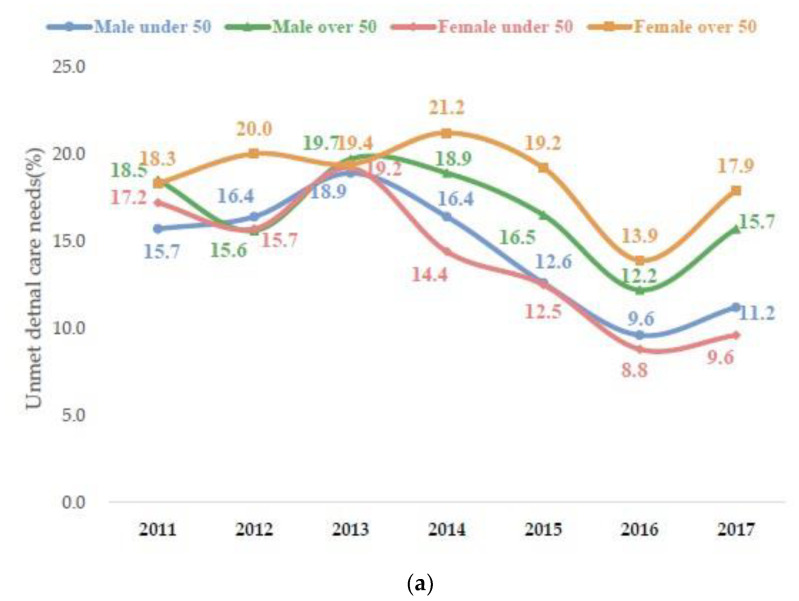

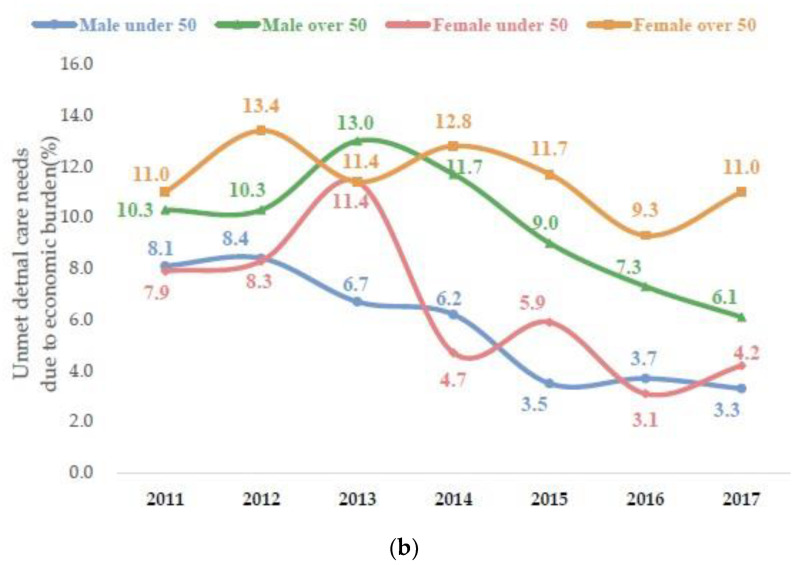
(**a**) Unmet dental care needs by gender and age group. (**b**) Unmet dental care needs due to economic burden by gender and age group.

Figure 2 presents the proportion of participants who experienced unmet dental care needs from 2011 to 2017. Unmet dental care needs have been witnessing frequent upward and downward trends. Specifically, unmet dental care needs did not decrease significantly in 2017, compared to 2011. Moreover, unmet dental care needs are distinct between those below and above 50 years of age. Particularly, the occurrence of unmet dental care needs has been caused due to different economic capabilities between men and women above 50 years of age.

### 3.2. Unmet Dental Care Needs Based on Socioeconomic Characteristics

Table 3 shows the classification of participants experiencing unmet dental care needs according to whether they were precarious workers. Concerning the difference in the occurrence of unmet dental care needs, significant differences were found among male workers based on their precarious employment (below 50 years: *p* = 0.001, above 50 years: *p* < 0.001), whereas no significant difference was found among women. Overall, regardless of gender and age, significant differences were found in unmet dental care needs because of economic burden across all groups, based on working status.

### 3.3. Unmet Dental Care Needs by Precarious Employment According to Gender and Age

Table 4 shows the effects of precarious employment, by gender and age group, on the occurrence of unmet dental care needs. For men over 50 years, those in Groups C and D were, respectively, 1.34 times (95% CI = 1.04–1.72) and 1.60 times (95% CI = 1.21–2.18) more likely to experience unmet dental care needs than those in Group A.

Concerning the causes of unmet dental care needs, owing to economic burden, men below 50 years in Group C were 1.74 times (95% CI = 1.17–2.57) more likely to experience unmet dental care needs. Owing to economic factors, the probability of having unmet dental care needs was 2.70 times higher (95% CI = 1.55–4.71) in Group D than in Group A. Men above 50 years in Groups B (OR = 3.36; 95% CI = 1.68–6.71), C (OR = 1.51; 95% CI = 1.06–2.16), and D (OR = 2.26; 95% CI = 1.54–3.33) were more likely to experience unmet dental care needs owing to economic factors than those in Group A.

For women below 50 years, unmet dental care needs owing to economic factors were 2.40 (95% CI = 1.40–4.11) and 3.04 (95% CI = 1.80–5.12) times higher in Groups C and D, respectively, compared to Group A. Among women above 50 years, those in Group D showed a 2.07 (95% CI = 1.15–3.74) times higher probability of experiencing unmet dental care needs owing to economic factors than those in Group A.

## 4. Discussion

In the present study, we aimed to identify the unmet dental care needs of Korean precarious workers using KHPS data from 2011 to 2017. Considering the characteristics of the Korean labor market, differences in results according to gender and age were also compared and analyzed.

The analyses of unmet dental care needs by gender and age group showed that male workers in Group D (job and income insecurity group) reported a higher rate of unmet dental care needs due to economic burden than those in Group A. Moreover, male workers in Group B (aged 50 years or over) were highly likely to experience unmet dental care needs owing to economic factors. This finding indicates that, regardless of employment relationship, unmet dental care needs are more likely to be experienced in low-income groups in South Korea. This may be due to the low coverage of dental sectors within the NHI system in Korea [20]. In Korea, health insurance coverage has increased for four major diseases (including cancer and cardiovascular disease), the older adult population, children, and disabled people, achieving an average of approximately 64% for the entire population and 74% for severe diseases, as of 2019. However, the dental sector still suffers from poor coverage (only 20%) [20,21]. The high rate of OOP payment in dental care services makes it difficult for low-income workers (Groups B and D) to use dental services. A previous study conducted in Australia on the effective mechanisms of financial barriers to the utilization of dental services also confirmed a direct impact on unmet medical care for low-income people. In particular, the results that the risk of avoided or delayed dental visit or prevented dental treatment by cost occurred higher in the 40s and 60s than in the younger age group and the female group supports the results of this study [22]. Hence, the Moon Care plan initiated by South Korean President Moon Jae-in recently announced that it will make efforts to strengthen various insurance coverages to reduce the OOP burden. These initiatives include cost-sharing and non-reimbursement by expanding public coverage for dental treatments such as neurotherapy and implants [23]. Therefore, it is necessary to strengthen effective healthcare coverage for dental services in the future.

We also found that female workers in Group D were more likely to have unmet dental care needs due to economic burden. In South Korea, female workers tend to be hired as regular workers when they enter the labor market. However, they often quit their jobs owing to marriage and childcare. Subsequently, they get re-employed in service sectors where both jobs and incomes become insecure (Group D) when they are 40–50 years old [24]. Most female workers are employed in the service sector, for example, as housekeepers or learning tutors. Thus, they can be easily replaced by others. They may face other challenges when searching for a new job, that may make it difficult for them to use dental services when necessary [25]. Therefore, it is necessary to ensure the job security of precarious female workers. The re-employment education program and employment support program also need to be considered.

The above results show that unmet dental care needs may be experienced because of employment insecurity alone, regardless of income level. The male workers and young female workers in Group C (e.g., construction daily workers and courier workers) also reported a higher rate of unmet dental care needs. According to previous studies, precarious employment leads to negative health consequences. In particular, many workers aged 50 years or older take up precarious employment or become self-employed in Korea, and often report that their physical health is threatened when they experience employment insecurity [26]. Workers with poor employment conditions are at a high risk of oral diseases due to the high possibility of unmet dental treatment as a result of preventive tooth access barriers [27,28,29].

Why did workers in Group C (e.g., temporary or daily wage workers at construction sites, freelance professionals, and courier workers) experience unmet dental care needs? The main reason is that sickness benefits have not been introduced in South Korea [19]. Workers with unstable employment may lose their income if they skip work to go for dental services visit. Therefore, the double burden of high health expenditure and income loss may further increase existing unmet dental care needs. As above-mentioned, in South Korea, some older workers (50 years old or over) leave their permanent jobs and start small-scale ventures and conduct their business either alone or with a few employees. However, when a hospital visit is necessary, they need to take a break from business and thus suffer economic losses [30]. This situation makes it difficult for workers in Group C to use dental services. Therefore, it is necessary to establish a sickness benefit system to compensate for the loss of income that occurs when workers are sick [30,31]. In June 2019, Korea introduced the “Seoul-type paid sick leave system,” that covered income loss only for inpatient services for vulnerable workers in Seoul, including contract workers [25]. However, the scope of the support was limited in terms of the duration, amount, and health services. Therefore, the national sickness benefit system must expand to comprehensively cover other areas and beneficiaries.

Unmet dental care needs for workers in Group C could have been caused by their working conditions, such as long working hours or night work. Most workers in Group C (e.g., construction daily workers and courier workers) work for more than 12 h a day to maintain a relatively high income [19]. A study highlighted that an association between working hours and unmet dental needs was observed in males [32]. This study suggested that unmet dental care needs are high because Korean hospitals close at 5 p.m. and workers usually finish their work at 7 p.m. [32]. Hence, longer working hours may lead to greater unmet dental care needs. A Japanese study revealed a link between overtime work and preventive dental visits in older male workers in their 40 s and 50 s [33]. Therefore, the 24 h dental clinic for overtime workers needs to be considered.

Although the present study reveals important findings, it has several limitations. First, in the process of classifying the participants, there were cases where the number of participants was small in a specific group (e.g., Groups B and D). Therefore, limitations in interpreting the study results for these groups were faced. Second, owing to the limitations of secondary data, the types and severity of diseases of the workers could not be considered. However, in this study, the subjective health status provided in the data was included in the analyses model as a control variable, when considering the overall health status of workers.

## 5. Conclusions

In conclusion, the results of this study highlight that South Korean workers experience a high degree of unmet dental care needs when employment and/or income are insecure. In particular, male workers aged above 50 years showed a high probability of unmet dental care needs because of economic factors. For women below 50 years, unmet dental care needs owing to economic factors were 2.40 and 3.04 times higher in Groups C and D. Therefore, policymakers should consider the type of work by gender and age groups to ensure adequate access to dental care. The findings suggest an urgent need to implement robust NHI policies to improve efforts aimed at reducing unmet dental care needs among precarious employees owing to financial factors. Furthermore, it is necessary to implement comprehensive labor market policies such as sickness benefits for precarious workers.

## Figures and Tables

**Table 1:** Definition of research groups.

		Paid Workers	Self-Employed
Job insecurity	(1) Employment contract	Temporary workers or daily employee	Self-employed with less than 4 employees and unpaid family workers
		Non-fixed-term temporary contract workers	
		Fixed-term contract workers	
	(2) Working hours	Part-time worker	
	(3) Employment relation	Indirect employment, Special employment	
Income insecurity	Income	Annual income less than two-thirds of the median income of all workers	
**Group**		**Example**	
Job and income security	Group A	e.g., permanent workers	
Job security with income insecurity	Group B	e.g., workers involved in cleaning, facility management, security, parking management, call centers, and public sectors	
Job insecurity with income security	Group C	e.g., temporary or daily wage workers in construction sites, freelance professionals, and courier workers	
Job and income insecurity	Group D	e.g., housekeepers, learning tutors, small-scale self-employed individuals with no or less than four employees	

**Table 2:** General characteristics of the study population from the Korea Health Panel Survey (2011–2017) ^†^.

		Male		Female		Total	
		n	%	n	%	n	%
Total		10,808	100.0	5985	100.0	16,793	100.0
Group	Group A	4253	39.4	1659	27.7	5912	35.2
	Group B	126	1.2	472	7.9	598	3.6
	Group C	4320	40.0	1560	26.1	5880	35.0
	Group D	2109	19.5	2294	38.3	4403	26.2
Marital status	Married	9649	89.3	4308	72.0	13,957	83.2
	Divorced or widowed	408	3.8	1010	16.9	1418	8.4
	Single	751	6.9	667	11.1	1418	8.4
Level of education	More than 12 years	4190	38.8	2030	33.9	6220	37.0
	Less than 12 years	6618	61.2	3955	66.1	10,573	63.0
Health Insurance status	Employee insured	7411	68.6	4391	73.4	11,802	70.2
	Self-employed insured	3338	30.9	1528	25.5	4866	29.0
	Medical aid	59	0.5	66	1.1	125	0.8
Social Insurance	Yes	4443	41.1	2715	45.4	7158	42.6
	No	6365	58.9	3270	54.6	9635	57.4
Private health insurance	Yes	8973	83.0	5423	90.6	14,396	85.7
	No	1835	17.0	562	9.4	2397	14.3
Self-rated health status	Good	5167	47.8	2651	44.3	7818	46.6
	Moderate	4870	45.1	2737	45.7	7607	45.3
	Poor	771	7.1	597	10.0	1368	8.1
Disability	No	10,253	94.9	5881	98.3	16,134	96.1
	Yes	555	5.1	104	1.7	659	3.9
Unmet dental care needs	No	9136	84.5	5003	83.6	14,139	84.2
	Yes	1672	15.5	981	16.4	2653	15.8
Unmet dental care needs due to economic burden	No	9136	84.5	5003	83.6	14,139	84.2
	Yes	808	7.5	518	8.7	1326	7.9

^†^ Weighted to population levels using sampling weights. Note: Based on employment and income security criteria, four groups were formed; (1) Group A comprised individuals with job and income security such as permanent workers, (2) Group B comprised individuals reporting job security with income insecurity, (3) Group C comprised individuals reporting job insecurity with income security, and (4) Group D comprised individuals with job and income insecurity such as precarious workers.

**Table 3:** Rates of unmet dental care needs according to the characteristics of Korean workers from the Korea Health Panel Survey (2011–2017) ^†^.

			18–49					Over 50				
			Yes	%	No	%	*p*-Value	Yes	%	No	%	*p*-Value
Male	Unmet dental care needs	Group A	360	12.4	2479	87.6	0.001	181	13.6	1233	86.3	<0.001
		Group B	10	20.1	34	79.9		15	19.9	67	80.1	
		Group C	286	15.8	1526	84.2		425	16.8	2083	83.2	
		Group D	53	20.1	187	79.9		342	19.7	1527	80.3	
	Unmet dental care needs due to economic burden	Group A	108	3.8	2479	96.2	<0.001	70	5.6	1233	94.4	<0.001
		Group B	6	11.4	34	88.6		14	19.4	67	80.6	
		Group C	116	7.0	1526	93.0		218	9.3	2083	90.7	
		Group D	33	13.0	187	87.0		243	14.0	1527	86.0	
Female	Unmet dental care needs	Group A	149	11.8	1085	88.2	0.053	62	14.8	362	85.2	0.055
		Group B	29	10.8	234	89.2		39	18.0	170	82.0	
		Group C	138	15.5	727	84.5		119	17.0	576	83.0	
		Group D	119	15.0	661	85.0		326	20.7	1188	79.3	
	Unmet dental care needs due to economic burden	Group A	34	3.0	1085	97.0	<0.001	19	4.8	362	95.2	<0.001
		Group B	12	5.1	234	94.9		15	8.0	170	92.0	
		Group C	65	8.3	727	91.7		66	9.8	576	90.2	
		Group D	79	10.1	661	89.9		228	15.2	1188	84.8	

^†^ Weighted to population levels using sampling weights. Note: Based on employment and income security criteria, four groups were formed: (1) Group A comprised individuals with job and income security such as permanent workers, (2) Group B comprised individuals reporting job security with income insecurity, (3) Group C comprised individuals reporting job insecurity with income security, and (4) Group D comprised individuals with job and income insecurity such as precarious workers.

**Table 4:** The unmet dental care needs by precarious employment on among Korean workers from the Korea Health Panel Survey (2011–2017) ^†^.

		Male				Female			
		18–49		Over 50		18–49		Over 50	
		OR	95% CI	OR	95% CI	OR	95% CI	OR	95% CI
Unmet dental care needs	Group A	1.00		1.00		1.00		1.00	
	Group B	1.55	0.65–3.68	1.53	0.82–2.86	0.91	0.55–1.50	1.19	0.70–2.01
	Group C	1.19	0.93–1.52	1.34	1.04–1.72	1.31	0.93–1.82	0.96	0.64–1.44
	Group D	1.47	0.96–2.25	1.60	1.21–2.18	1.33	0.96–1.85	1.07	0.73–1.58
Unmet dental care needs due to economic burden	Group A	1.00		1.00		1.00		1.00	
	Group B	2.20	0.78–6.21	3.36	1.68–6.71	1.58	0.67–3.72	1.59	0.70–3.60
	Group C	1.74	1.17–2.57	1.51	1.06–2.16	2.40	1.40–4.11	1.59	0.86–2.93
	Group D	2.70	1.55–4.71	2.26	1.54–3.30	3.04	1.80–5.12	2.07	1.15–3.74

^†^ Weighted to population levels using sampling weights. Note: Based on employment and income security criteria, four groups were formed: (1) Group A comprised individuals with job and income security such as permanent workers, (2) Group B comprised individuals reporting job security with income insecurity, (3) Group C comprised individuals reporting job insecurity with income security, and (4) Group D comprised individuals with job and income insecurity such as precarious workers.

## Data Availability

Not applicable.
